# Effect of Sintering Factors on Properties of Al-Rich PTFE/Al/TiH_2_ Active Materials

**DOI:** 10.3390/polym13111705

**Published:** 2021-05-23

**Authors:** Yilei Wang, Chunlan Jiang, Zaicheng Wang

**Affiliations:** State Key Laboratory of Explosion Science and Technology, Beijing Institute of Technology, Beijing 100081, China; wylbitDr@163.com (Y.W.); wangskyshark@bit.edu.cn (Z.W.)

**Keywords:** Al-rich PTFE/Al/TiH_2_ active material, sintering process factors, quasi- static compression test, phase analysis, microstructure, mechanical properties

## Abstract

Sintering process is an important part of the specimen preparation process, which directly affects the properties of materials. In order to obtain the best sintering control factors of Al-rich PTFE/Al/TiH_2_ active materials, Al-rich PTFE/Al/TiH_2_ active specimens with different sintering control factors were prepared using a mold pressing sintering method. A quasi-static compression experiment was carried out on a universal material testing machine, and a real stress-strain curve was obtained. The effects of sintering control factors on the properties of Al-rich PTFE/Al/TiH_2_ active materials were analyzed by means of mechanical parameters such as compressive strength, failure strain and toughness. SEM and XRD were used to analyze the microstructure and phase of the sintered samples. The results show that: (1) With the increase of cooling rate, the density, yield strength, strain hardening modulus, compressive strength and toughness of Al-rich Al/PTFE/TiH_2_ specimens decrease gradually, while the failure strain and pores of the specimens increase gradually. (2) With the increase of sintering temperature, the density, maximum true strain and toughness of the specimens first increase and then decrease, and the failure strain of the specimens gradually increases. When the sintering temperature is 360 °C, the PTFE matrix and particles inside the specimen are closely combined, a small number of particles are exposed on the PTFE matrix and there are a small number of voids. (3) With the increase of holding time at 360 °C, the strength and toughness of the material first decrease and then increase. When the holding time is 6 h, the interface between particles and matrix inside the specimen is the strongest, and the crack propagation inside the specimen is less. (4) When the sintering time increased from 1 h to 4 h at 315 °C, the compressive strength of the specimen increased by 1.62%, the toughness of the specimen decreased by 0.55% and the failure strain of the specimen decreased by 0.54%. The interface between PTFE matrix and particles is the strongest and the crack propagation is less in the specimen with a holding time of 4 h. (5) Above all, the optimum sintering parameters of Al-rich Al/PTFE/TiH_2_ materials are cooling rate of 25 °C/h, sintering temperature of 360 °C, holding time of 6 h and holding time of 4 h at 315 °C. (6) The reactivity of Al-rich Al/PTFE/TiH_2_ specimens with 10% content of TiH_2_ under static compression is not significantly affected by sintering parameters.

## 1. Introduction

Polytetrafluoroethylene (PTFE) is a kind of fluorine-containing polymer, with high a fluorine content of over 70%, which is characterized by a low friction coefficient, acid and alkali resistance, high and low temperature resistance, non-combustibility, non-stickiness and lubricity. It is often mixed with an active metal or metal oxide filler via a special process to make an active composite with a certain strength, hardness, insensitivity and other properties. As an important branch of energetic materials abroad, active composites are known as reaction materials, and are also known as impact induced energetic materials. Under the action of high impact load, a large amount of chemical energy can be released between the components of the material or between the material and the environment.

**Figure 1:**
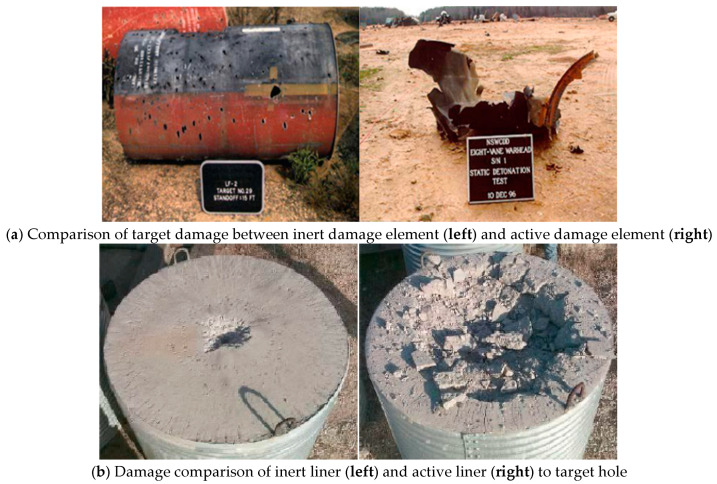
The damage effects of inert damage element and active damage element on target hole and interior are compared.

Metal/PTFE active composite (abbreviation MPRC) is a new type of advanced material of this kind of impact-initiated energetic material. MPRC has a wide application prospect in the military and civil fields. In the military field, MPRC can replace inert damage element material without exothermic reaction characteristics in existing weapons. It can add high thermal energy of reaction on the basis of kinetic energy and has secondary reaction effects such as deflagration and explosion, so as to greatly improve the damage effect of weapons. In 2011, the US Navy Surface Warfare Center demonstrated the effectiveness of “high density reactive composites” (HDRC). The density of HDRC is equivalent to that of steel, the strength of HDRC reaches the level of aluminum alloy, and the energy of HDRC is 1.5 times that of TNT [1]. Therefore, MPRC can be made into active damage element, which can be used in air defense missiles [2] or large area soft kill weapons [3] to carry out kinetic energy penetration and high-energy explosion [4] on hard targets such as armor, concrete and warships, thus increasing the damage effect of the penetration hole and targeting the interior. The damage effects of inert damage element and active damage element on target hole and interior are compared, as shown in Figure 1. In addition, the micro ignition device fabricated by MPRC can save the complex structure [4,5] of warhead fuze. In the civil field, the perforating charge prepared by MPRC can increase the production efficiency of oil or shale gas [6,7] and reduce the production cost [8,9]. MPRC can also be used as a conductor material for initiating devices [10,11] and for low-temperature synthesis of high melting point ceramic powders [12].

Al/PTFE is one of the most representative reactive materials of MPRC. Compared with traditional explosives, propellants and pyrotechnics, it has a higher energy release level, higher safety and better physical and chemical properties. Therefore, this material is one of the best choices for domestic and foreign scholars to carry out experimental research in recent years. In the last ten years, the main research based on Al/PTFE reaction materials is as follows: (1) study on the composition ratio and mixing methods of Al/PTFE-based active materials; (2) study on the mechanical properties, reaction characteristics, impact sensitivity, ignition mechanism and hot spot formation mechanism of crack tip of Al/PTFE-based active materials; (3) study on the energy release characteristics of Al/PTFE-based active materials; (4) study on the establishment of a theoretical model of impact-induced chemical reaction of Al/PTFE-based active materials. Among them, some scholars introduce high density metals such as Ni [13], W [14,15] and Ta [16] into Al/PTFE-based active materials to improve their hardness, density, strength and other characteristics; some scholars have added metal oxides CuO [17], Fe_2_O_3_ [18] and MnO_2_ [19] to Al/PTFE-based active materials to improve their reaction characteristics; some scholars have added high-energy additives such as TiH_2_ [20] and ZrH_2_ [21] to Al/PTFE-based active materials to improve their energy release characteristics. Based on published literature of the above studies, it can be found that most scholars mainly study the influence of distribution ratio, particle size and other factors on material properties through a given sintering control curve, while few scholars study the influence of sintering control factors on material properties. In particular, the research on the influence of sintering control factors on the properties of active composites is blank.

Sintering process is an important link in the production of material specimens, which directly affects the sintering degree of the active specimens, and then it is particularly important to improve the density, hardness, strength and other properties of the material specimens. It also affects the mechanical properties, reaction characteristics, sensitivity, terminal damage effect and energy release of the material. Therefore, in this paper, the quasi-static compression experiments of Al-rich Al/PTFE/TiH_2_ active materials were carried out using four sintering control factors (cooling rate, sintering temperature, holding time at 360 °C and holding time at 315 °C). The influence rule of four control factors on the microstructure and mechanical properties of Al-rich Al/PTFE/TiH_2_ were analyzed. The phase analysis of Al-rich Al/PTFE/TiH_2_ specimens after sintering was carried out via X-ray diffraction (XRD) to detect whether the reaction occurred.

## 2. Materials and Methods

### 2.1. Theoretical Maximum Density and Actual Density of Al-Rich Al/PTFE/TiH_2_ Specimens

The theoretical maximum density (TMD) of Al-rich Al/PTFE/TiH_2_ specimens can be calculated using the following formula.

(1) $$\rho_{\mathrm{TMD}}=\frac{1}{\frac{w_{\mathrm{Al}}}{\rho_{\mathrm{Al}}}+\frac{w_{\mathrm{PTFE}}}{\rho_{\mathrm{PTFE}}}+\frac{w_{\mathrm{Ti}H_{2}}}{\rho_{\mathrm{Ti}H_{2}}}}$$

where w_Al_, w_PTFE_ and $w_{\mathrm{Ti}H_{2}}$ are the mass fractions of Al, PTFE and TiH_2_, respectively; ρ_Al_, ρ_PTFE_ and $\rho_{\mathrm{Ti}H_{2}}$ are the densities of Al, PTFE and TiH_2_, respectively.

Assuming that the mass of the pending specimen in the air is m_1_, the pending specimen is suspended using a gravimeter and placed in a beaker with water and does not touch the wall of the beaker, the measured gravity is N, the volume of the pending specimen is V, the corresponding density of water at ambient temperature is ρ_water_, and the local acceleration of gravity is g_local_. According to Archimedes’ principle [22], the actual density of the specimens can be obtained as follows:

(2) $$\rho_{\mathrm{true}}=\frac{m_{1}}{V}=\frac{m_{1}}{\frac{m_{1}g_{\mathrm{local}}-N}{\rho_{\mathrm{water}}g_{\mathrm{local}}}}=\frac{m_{1}\rho_{\mathrm{water}}g_{\mathrm{local}}}{m_{1}g_{\mathrm{local}}-N}$$

### 2.2. Sample Preparation

According to the literature published by Zhongshen Yu [20], Al/PTFE/TiH_2_ active material containing 10% content of TiH_2_ has moderate energy release rate and reaction threshold. Therefore, the Al-rich PTFE/Al/TiH_2_ active material with a mass ratio of 40/50/10 was used as the specimen material of this experiment. Table 1 presents information and the mass ratio of components of Al-rich PTFE/Al/TiH_2_ active material.

**Figure 2:**
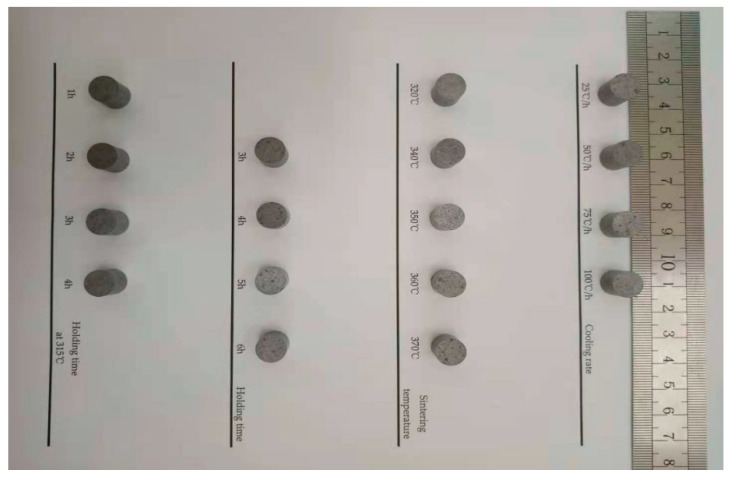
Physical diagram of Al-rich Al/PTFE/TiH_2_ active specimen.

**Figure 3:**
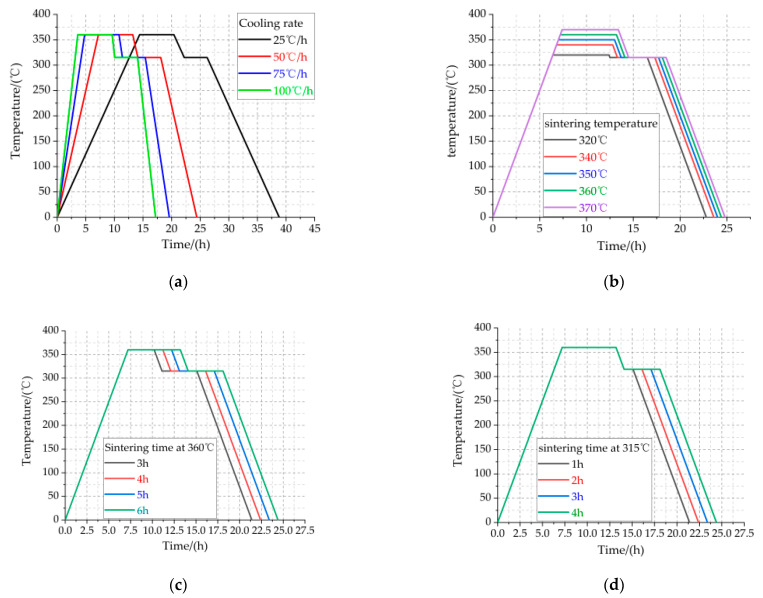
Sintering process curve under four control factors. (**a**) Cooling rate sintering process curve; (**b**) Sintering temperature sintering process curve; (**c**) Holding time at 360 °C sintering process curve (**d**) Holding time at 315 °C sintering process curve.

The complete process of preparing Al-rich Al/PTFE/TiH_2_ specimens is as follows:

1. According to the mass ratio of 40/50/10, Al powder, PTFE powder and TiH_2_ powder were weighed using an electronic scale and mixed in a beaker. At this time, an appropriate amount of anhydrous ethanol was added into the beaker while continuously stirring, and the approximate fully mixed solution was made for about 30 min. The beaker containing the mixed solution was dried in a vacuum drying oven at 55 °C for 48 h, and a fully mixed bulk Al/PTFE/TiH_2_ solid mixture was obtained.
2. The bulk Al/PTFE/TiH_2_ solid mixture was crushed with a glass rod, and continuously stirred to powder state, The powder was pressed into a columnar embryo with different preforming pressures and holding times using a hydraulic press and forming mold.
3. In the sintering furnace with argon atmosphere, the columnar embryo was sintered according to the sintering process curves under the following four sintering factors. The sintered columnar embryo was placed at room temperature for 2 days to eliminate the internal stress. Finally, Al-rich Al/PTFE/TiH_2_ specimens with a size of Φ10 mm × 10 mm were obtained via machining. Figure 2 is the physical diagram of Al-rich Al/PTFE/TiH_2_ active specimens prepared under four control factors. Figure 3 shows the control curve of the sintering process under the four control factors.

The sintering parameters shown in Figure 3a–d are listed in Table 2, Table 3, Table 4 and Table 5, respectively. Cooling rate, being the same as heating rate, could be referred to as “ramp rate”.

### 2.3. Experimental Contents

**Figure 4:**
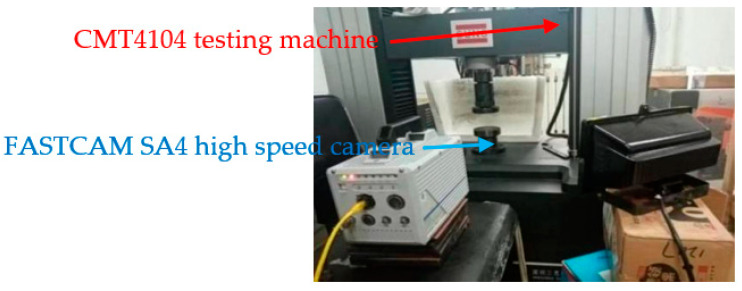
Layout of quasi-static compression experiment.

The experimental arrangement is shown in Figure 4. The quasi-static compression test was carried out on 13 groups of experimental specimens using a CMT4104 microcomputer-controlled electronic universal testing machine. The experimental loading strain rate $\dot{\varepsilon }$ is 0.1 /s, the corresponding pressure head uniform pressing rate is 60 mm/min, the maximum experimental force is 50 kN, the accuracy level is 0.5, the voltage is 220 V, and the power is 0.4 kW. In order to obtain stable and reliable data, at least three repeated experiments were carried out on each group of experimental specimens. Before the experiment, in order to reduce the friction between the indenter and the end of the specimen and help the transverse deformation of the end face of the specimen, a proper amount of Vaseline was applied on the end face of the specimen. All experiments were carried out at room temperature. The compression process of the test specimen was recorded using FASTCAM SA4 high-speed camera. The shooting speed is 50 fps, the resolution is 512 × 512 ppi, and the starting trigger point is used to capture. A FEINOVA450 scanning electron microscope (SEM) was used to analyze the microstructure of the bottom surface of the specimen. A Rigaku smartlab9 X-ray diffractometer (XRD) was used for phase analysis of the sintered specimens.

**Figure 5:**
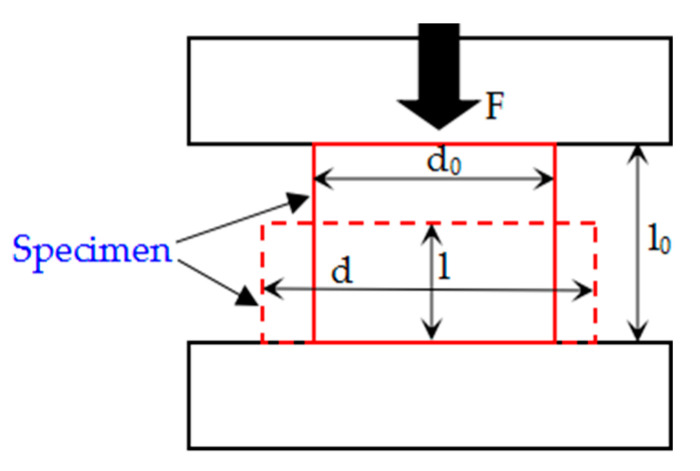
Deformation diagram of experimental specimen during quasi-static compression.

In order to facilitate the transformation between the engineering stress and strain and the real stress and strain, it can be considered that all experimental specimens always keep the cylindrical shape and volume unchanged during the compression process, and the deformation and size of the specimens are shown in Figure 5. The true strain and true stress can be obtained as follows:

(3) $$\left\{ \begin{array}{l} \varepsilon_{\mathrm{true}}=\int_{l}^{l_{0}}d\varepsilon =\int_{l}^{l_{0}}\frac{\mathrm{dl}}{l}=\ln\frac{l_{0}}{l}=\ln\frac{1}{1-\varepsilon_{\mathrm{eng}}}=-\ln\left(1-\varepsilon_{\mathrm{eng}}\right)\\ \sigma_{\mathrm{true}}=\frac{F}{S}=\frac{\mathrm{Fl}}{\pi {\left(\frac{d_{0}}{2}\right)}^{2}l_{0}}=\frac{\frac{\pi d_{0}^{2}\sigma_{\mathrm{eng}}}{4}l}{\pi {\left(\frac{d_{0}}{2}\right)}^{2}l_{0}}=\frac{\sigma_{\mathrm{eng}}l}{l_{0}}=\sigma_{\mathrm{eng}}\left(1-\varepsilon_{\mathrm{eng}}\right) \end{array}\right.$$

d_0_, l_0_, l, F, S, d, σ_eng_ and ε_eng_ are the original diameter, original height, instantaneous height, instantaneous loading stress, instantaneous area, instantaneous diameter, engineering stress and engineering strain of the specimen, respectively. Combined with the Formulas (1)–(3), the theoretical maximum density, actual density and real stress-strain data of Al-rich Al/PTFE/TiH_2_ specimens under sintering control factors, can be obtained. 

## 3. Results and Discussion

### 3.1. Influence of Cooling Rate

**Figure 6:**
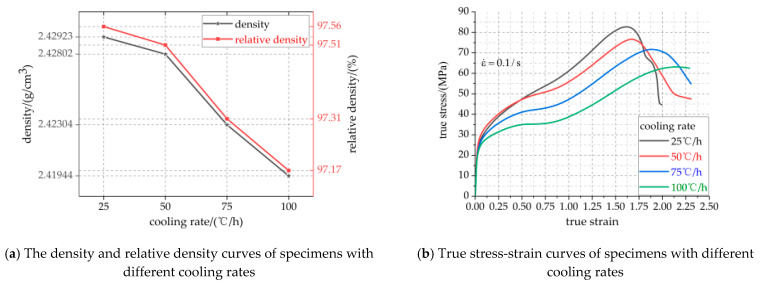

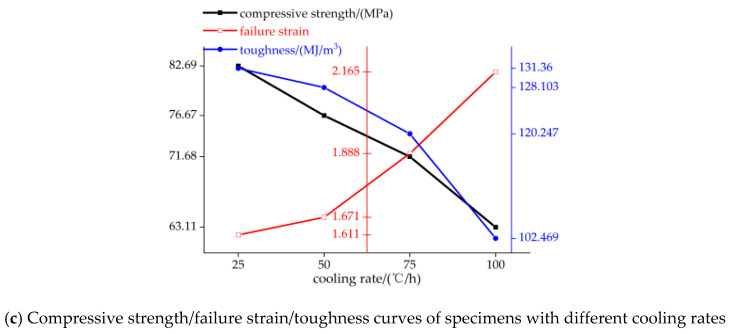
Visualization curves of related parameters of specimens with different cooling rates.

The cooling rate determines the crystallization rate of PTFE matrix, which affects the crystallinity of PTFE matrix and further affects the properties of PTFE-based active materials. The density and relative density curves, true stress-strain curves and compressive strength/failure strain/toughness curves of specimens with different cooling rates are shown in Figure 6a–c, respectively. The specific parameters of Figure 6a,c are listed in Table 6.

It can be seen from Figure 6a and Table 6 that the cooling rate has a significant effect on the density of Al-rich Al/PTFE/TiH_2_ active material specimens. With the increase of cooling rate, the density and relative density of the specimen decrease gradually. When the cooling rate is 25 °C/h, the maximum density of the active specimen is 2.42923 g/cm^3^, which is 97.56% of the theoretical maximum density (TMD). When the cooling rate is 100 °C/h, the minimum density of the active specimen is 2.41944 g/cm^3^, 97.17% of which is the theoretical maximum density. Therefore, the density of the specimen with a cooling rate of 25 °C/h is the largest and increases by 0.4% compared with other specimens. The analysis shows that when the temperature is in the range of 310–315 °C, the crystallization rate of macromolecules in PTFE matrix is faster. With the increase of cooling rate, the less the residence time in this temperature range and the smaller the degree of macromolecular crystallization, and the smaller the crystallinity, the smaller the material density.

It can be seen from Figure 6b,c and Table 6 that the four groups of specimens have experienced three stages of elastic deformation, plastic deformation and strain fracture failure. The elastic modulus of the four groups of specimens is basically the same, because the four groups of specimens have the same composition and content of PTFE, and the elastic modulus is mainly borne by the amorphous region in the amorphous form of PTFE matrix [23]. After elastic deformation, the four groups of specimens show strain hardening phenomenon, but their mechanical properties are significantly affected by the cooling rate. With the increase of cooling rate, the yield strength, strain hardening modulus, compressive strength and toughness of the specimen decrease gradually, and the failure strain of the specimen increases gradually. Compared with the specimens with other cooling rates, the yield strength, strain hardening modulus, compressive strength and toughness of the specimens with a cooling rate of 25 °C/h increased by 23.08%, 26.32%, 23.68% and 21.99%, respectively, and the failure strain of which decreased by 25.59%. This also confirmed that the compressive strength of the material can be improved by slow cooling as described in reference [24]; this is because with the increase of cooling rate, PTFE molecules have no time to crystallize, resulting in the decrease of crystallinity and the heat and temperature required for crystallization and melting. The maximum true strain of the four groups of specimens is more than 2, and they all have good ductility. Based on the above knowledge, the overall mechanical properties of specimens with a cooling rate of 25 °C/h are better. 

**Figure 7:**
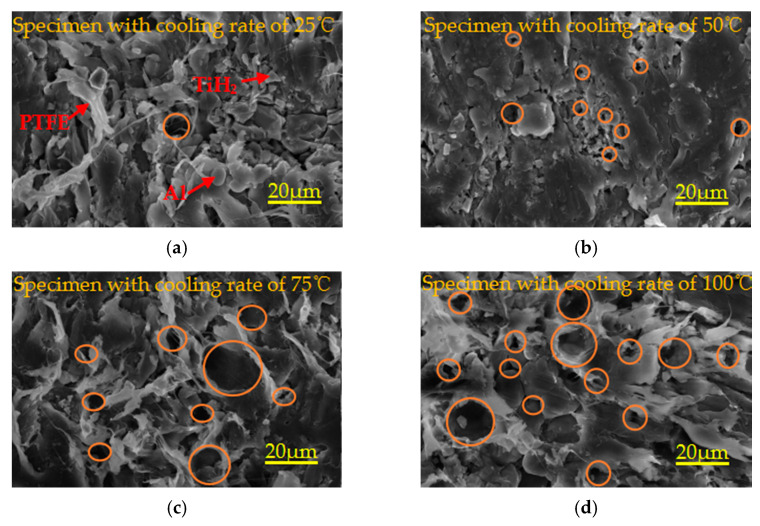
Microstructure of specimens with different cooling rates. (**a**–**d**) are the microstructures of specimens with cooling rates of 25 °C/h, 50 °C/h, 75 °C/h and 100 °C/h, respectively.

Figure 7 shows the micro morphology of the specimens sintered at different cooling rates, and the chocolate yellow circles show the voids. With the increase of cooling rate, the time for the PTFE matrix inside the specimen to decrease from 360 °C amorphous melting state to 315 °C cold crystallization melting state decreases gradually, which creates the phenomenon of an increasing vibration amplitude of PTFE matrix atoms and gradually decreasing particle atoms and also the phenomenon of irregular diffusion of PTFE matrix atoms and gradually weakening particle atoms, resulting in the number of internal voids filled by the PTFE matrix molten state gradually decreasing. Therefore, the crystalline surface of the PTFE matrix decreases and the void increases gradually. Before sintering, the accumulation order of PTFE matrix and particles inside the specimen is established via an external force, which will be destroyed due to the thermal movement of molecules during sintering. At the end of sintering, there is shrinkage of the PTFE phase change crystal volume and the internal stress increases, and the voids and other structural defects appear during cooling. It can be seen from Figure 7 that with the increase of cooling rate, the number of voids in the specimen increases gradually, the cohesion between the PTFE and particles decreases, the crystallinity decreases gradually, and the crystalline volume shrinkage of PTFE increases gradually. This is because with the increase of cooling rate, the crystalline volume of the PTFE matrix has no time to shrink. Therefore, the above findings can be used as a powerful explanation for the decrease of mechanical properties such as strength and density of materials with the increase of cooling rate.

### 3.2. Influence of Sintering Temperature

**Figure 8:**
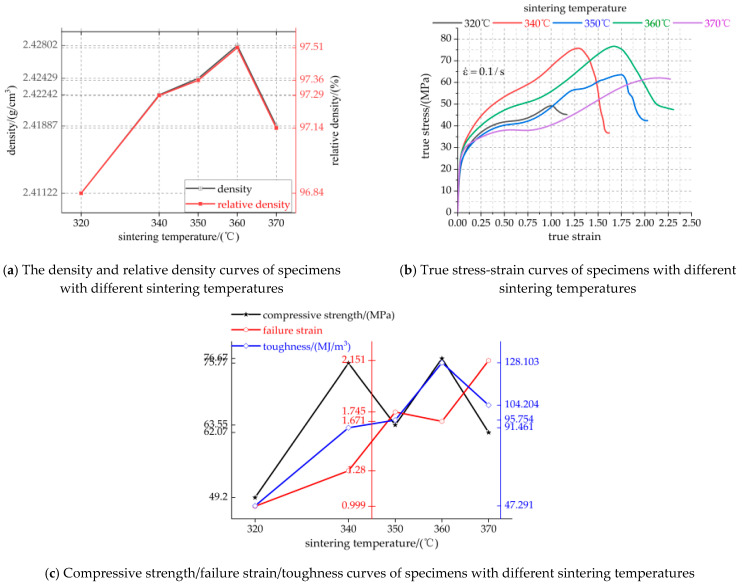
Visualization curve of relevant parameters of specimens with different sintering temperature.

The density and relative density curves, true stress-strain curves and compressive strength/failure strain/toughness curves of specimens with different sintering temperatures are shown in Figure 8a–c, respectively. The specific parameters of Figure 8a,c are listed in Table 7.

It can be seen from Figure 8a and Table 7 that with the increase of sintering temperature, the density and relative density of the specimen first increase and then decrease. When the sintering temperature is 360 °C, the maximum density of the specimen is 2.42802 g/cm^3^, which is 97.51% of the theoretical maximum density (TMD). When the sintering temperature is 320 °C, the density of the specimen is the lowest, which is due to the fact that the sintering temperature is lower than the crystallization and melting temperature of 327 °C of polytetrafluoroethylene (PTFE), which leads to the same crystalline morphology of the sintered sample as that of PTFE raw material powder [25]. When the temperature is above 327 °C, the PTFE matrix inside the specimen is in the state of molten expansion flow, resulting in the fully covered contact between the PTFE molten matrix and the particles. When the sintering temperature is higher than 360 °C, the density of the specimen begins to decrease, which may be caused by the abnormal change of the crystallinity of the specimen due to the excessively high sintering temperature, which leads to the “overburning” phenomenon of the specimen [26]. 

It can be seen from Figure 8b,c and Table 7 that the five groups of specimens satisfy Hooke’s law in the elastic section and almost show the same properties. The elastic modulus is very large and almost the same. This is because the specimens contain the same PTFE matrix, Al and TiH_2_ particles, and these particles are within the elastic limit at this stage. The strength, maximum true strain, failure strain and toughness of the specimen sintered at 320 °C are the minimum, which are 49.20 MPa, 1.166, 0.999 and 47.291 MJ/m^3^, respectively. This is because the PTFE matrix inside the specimen sintered at 320 °C is in the state of melting without recrystallization, which leads to the worst mechanical properties of the specimen. The maximum true strain of the specimens with sintering temperature above 350 °C is over 2. Therefore, the specimens with sintered temperatures above 350 °C have good ductility. The compressive strength of specimens sintered at 350 °C and 370 °C is very low, 63.55 MPa and 62.07 MPa, respectively. There may be three reasons for this situation: one is that the density distribution of the specimen is not uniform in the process of pressing the specimen; the other is that the viscosity of PTFE matrix is low, the high-temperature melting fluidity is good, and it is easy to produce many concentrated shrinkage cavities with a smooth inner wall and small volume; the third is that the adhesion between PTFE matrix and particles is poor, and it is easy to produce tiny cracks under pressure. With the increase of sintering temperature, the maximum true strain and toughness of the specimen first increase and then decrease, and the failure strain increases gradually. The compressive strength, maximum true strain and toughness of the specimens sintered at 360 °C are the largest, 76.67 MPa, 2.306 and 128.103 MJ/m^3^, respectively. In conclusion, the mechanical properties of the specimen sintered at 360 °C are the best.

**Figure 9:**
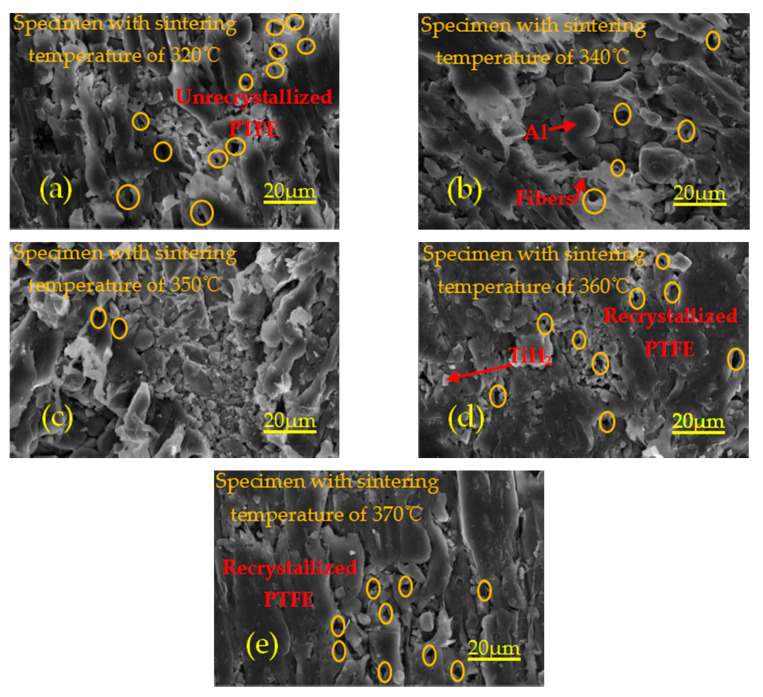
Local microtopography of specimens with different sintering temperatures under the same magnification. (**a**–**e**) are the microstructures of specimens with sintering temperature of 320 °C, 340 °C, 350 °C, 360 °C and 370 °C, respectively.

Figure 9 shows the microstructure of specimens sintered at different temperatures. Orange circles denote pores. As can be seen from Figure 9a, due to the sintering temperature of 320 °C, lower than the melting temperature of 327 °C of PTFE crystallization, only a small number of Al particles and TiH_2_ particles are wrapped in the PTFE matrix inside the specimen after heating, expansion, flow and non-recrystallization cooling, and most of the particles are exposed on the PTFE matrix. Therefore, the bonding between PTFE matrix and particles is not very tight, and there are a certain amount of pores with different volumes. As can be seen from Figure 9b,d, most of the particles are embedded in the recrystallized PTFE matrix, and a small number of particles are exposed on the PTFE matrix. The PTFE matrix is tightly bound to the particles, and there are a lot of pores. It can be seen from Figure 9c,e that most of the particles are exposed on the PTFE matrix, and there are a lot of pores. The phenomenon of pore formation may be due to the excessive residual stress released at high temperature during sintering, which leads to the existence of many pores inside the specimen. Therefore, excessive porosity is an important factor to decrease the strength of materials.

### 3.3. Influence of Holding time at 360 °C

**Figure 10:**
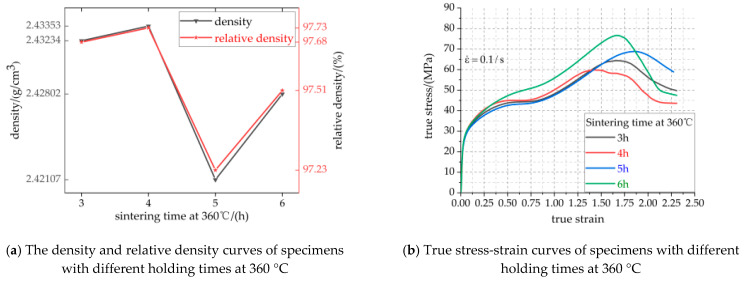

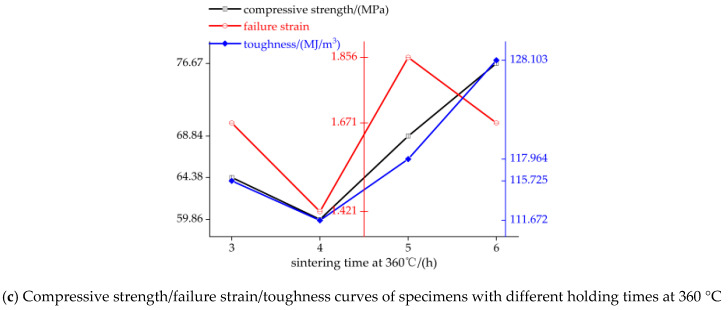
Visualization curve of mechanical property parameters of specimens with different holding time at 360 °C.

The density and relative density curves, true stress-strain curves and compressive strength/failure strain/toughness curves of specimens with different holding times at 360 °C are shown in Figure 10a–c, respectively. The specific parameters of Figure 10a,c are listed in Table 8.

It can be seen from Figure 10a and Table 8 that the density and relative density of the specimen are significantly affected by the holding time at 360 °C. The maximum density of the specimens is 2.43353 g/cm^3^ when the holding time is 4 h, which is 0.512% higher than that of the samples with a holding time of 5 h. When the holding time is more than 5 h, the density of the specimen does not decrease but increases, which may be due to the fact that too long a holding time aggravated the decomposition of the PTFE molecular chain and decreased the molecular weight. The smaller the entanglement between and within molecules, the stronger the activity of the molecular chain and the stronger the crystallization ability, which leads to the increase of crystallinity [27,28]. It can be seen from Figure 10b,c and Table 8 that the specimens with holding times of 3 h, 4 h, 5 h and 6 h at 360 °C respectively experienced elastic-plastic deformation, yield, strain hardening and fracture failure during static compression [29], and the deformation of the specimens was relatively large. As the specimen has the same composition and content of PTFE, the four groups of specimens have the same elastic modulus in the elastic section. In the stage of strain hardening and fracture failure, the influence of holding time at 360 °C is remarkable, which can be reflected by the main mechanical parameters such as compressive strength, failure strain and toughness. With the increase of holding time at 360 °C, the strength and toughness of the material first decrease and then increase. The strength and toughness of the specimens with a holding time of 6 h are the largest, 76.67 MPa and 128.103 MJ/m^3^, respectively, increased by 21.93% and 12.83%, respectively, compared with those of the specimen with a holding time of 4 h. The maximum true strains of the four groups of specimens discussed above are all above 2.25, which indicates that they have good ductility. In conclusion, the mechanical properties of the specimens with a holding time of 6 h at 360 °C are the best.

**Figure 11:**
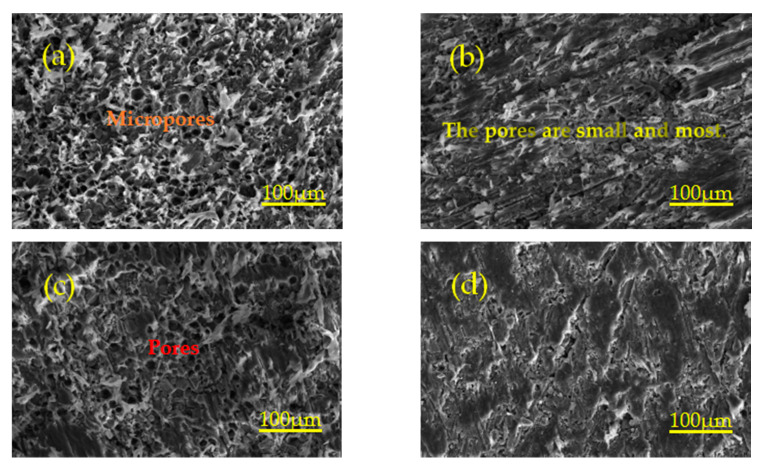
Microstructure of the specimen with different holding time at 360 °C. (**a**–**d**) are the microstructures of specimens with holding times of 3 h, 4 h, 5 h and 6 h at 360 °C, respectively.

Holding time affects the filling process of molten PTFE fluid in the voids between particles and the spreading process of Al powder and TiH_2_ powder surface, thus resulting in the formation of different micro morphology. Figure 11a–d show the same magnification micromorphology image of the specimen with holding times of 3 h, 4 h, 5 h and 6 h, respectively. Due to the same preforming pressure and the pressure holding time before sintering, there is approximately the same amount of void inside the specimen. Short time heat preservation makes PTFE fluid not fully fill the internal voids of the specimen, which leads to many tiny pores being generated inside the specimen, and then forms a weak strength interface between PTFE matrix and particles. With the increase of holding time, the PTFE fluid has enough time to maintain the molten flow state, which can fully fill a large number of voids inside the specimen and make the PTFE matrix tightly bond with the particles to form a high-strength interface. The high-strength interface can transfer the load from the PTFE matrix to the particles and can also hinder the crack propagation direction in PTFE matrix. Therefore, the material has strong mechanical properties. Similarly, the weak strength interface is not conducive to the transfer of load between particles and is easy to crack propagation in PTFE matrix. Therefore, the material has weak mechanical properties. It can be seen from Figure 11 that Al and TiH_2_ particles are embedded in the PTFE matrix, forming a composite interface between the PTFE matrix and particles. The strength and weakness of the interface can affect the crack propagation. The strong interface can hinder crack propagation, while the weak interface is easy to crack propagation. In Figure 11b, the interface between particles and matrix is the weakest, and the cracks propagate a lot, and the overall mechanical properties of the specimen are the worst. In Figure 11d, the interface between particles and matrix is the strongest, and the crack propagation is less, and the overall mechanical properties of the specimen are the best.

### 3.4. Influence of Holding Time at 315 °C

**Figure 12:**
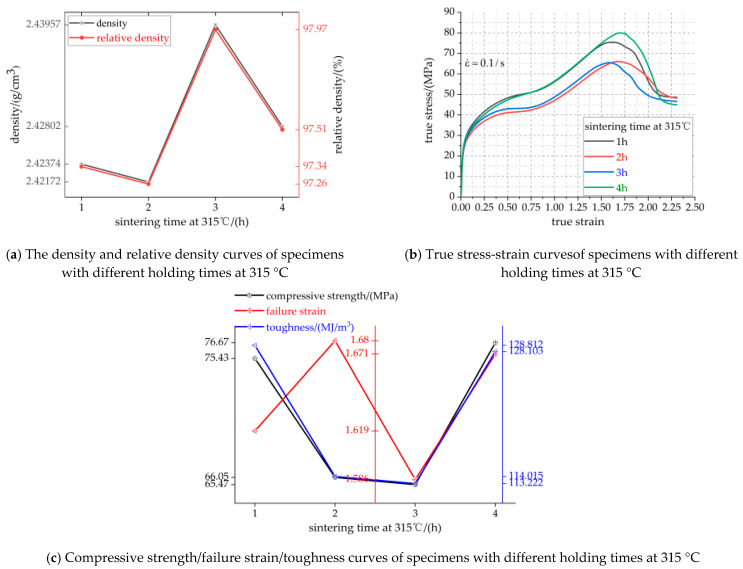
Visualization curve of mechanical properties parameters of specimens with different sintering time at 315 °C.

The density and relative density curves, true stress-strain curves and compressive strength/failure strain/toughness curves of specimens with different holding times at 315 °C are shown in Figure 12a–c, respectively. The specific parameters of Figure 12a,c are listed in Table 9.

It can be seen from Figure 12a and Table 9 that with the increase of holding time at 315 °C, the maximum density of the specimen with a holding time of 3 h is 2.43957 g/cm^3^, reaching 97.97% of the theoretical maximum density (TMD). It can be seen from Figure 12b,c and Table 9 that the overall mechanical properties of the specimens with holding for 2 h and 3 h are the worst. The four groups of specimens have an elastic stage, strain hardening stage and fracture failure stage. When the sintering time at 315 °C increased from 1 h to 4 h, the compressive strength of the specimens decreased from 75.43 MPa to 65.47 MPa, and then increased to 76.67 MPa, an increase of 1.62%. The toughness of the specimens decreased from 128.812 MJ/m^3^ to 113.222 MJ/m^3^, then increased to 128.103 MPa, a decrease of 0.55%. The failure strain of the specimens increased from 1.619 to 1.68 and then decreased to 1.671, a decrease of 0.54%. The maximum true strain of the four groups of specimens is more than 2.25, which demonstrates good ductility. The strength and strain loss of the specimens with a holding time of 4 h are the largest, the toughness of which is only 0.55%, smaller than that of specimen with holding for 1 h. It may be too short a heating time, which makes the movement of the molecular chain of PTFE weaken, and the degree of decomposition of the macromolecular chain into a small molecular chain is reduced. The decrease of the molecular chain reduces the binding tightness between the PTFE matrix and particles, and the internal defects increase. The overall mechanical properties of the specimen with a holding time of 4 h at 315 °C are the best. 

**Figure 13:**
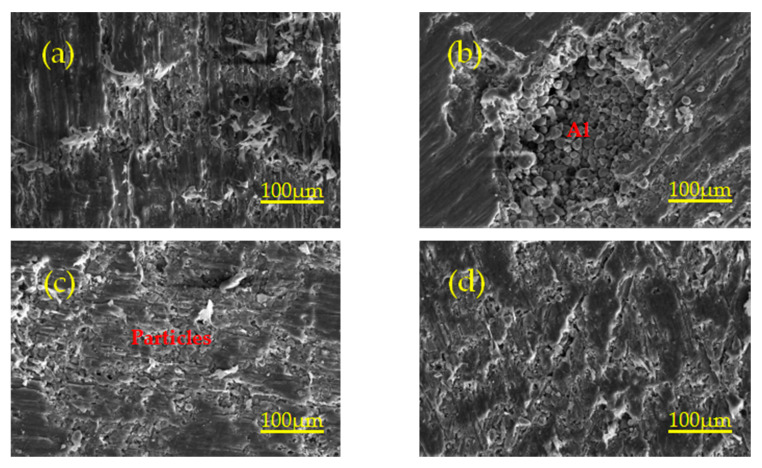
Micromorphology of specimens with different holding time at 315 °C. (**a**–**d**) are the microstructures of specimens with holding times of 1 h, 2 h, 3 h and 4 h at 315 °C, respectively.

Figure 13a–d show the same magnification microscopic morphology image of the specimens with holding times of 1 h, 2 h, 3 h and 4 h at 315 °C. 

The main function of sintering is to obtain the cross-linking of polymer matrix and to fuse the particles with PTFE matrix to form a composite with certain strength and hardness. If the crystallinity of materials is different, the strength is also different. Because the sintering temperature of 315 °C can make the crystallization speed of PTFE matrix the fastest, it can be kept at this temperature for a certain time to improve the crystallinity of the material, and then improve the strength and other mechanical properties of the material. The degree of crystallinity of PTFE matrix can be qualitatively judged by observing the micro factors such as voids, pores and the coating integrity of PTFE matrix and particles, so as to infer whether the mechanical properties of the specimen are good or bad. In Figure 13a,d, the bond tightness between PTFE matrix and particles is higher than that in Figure 13b,c. In Figure 13b,c, a large number of Al and TiH_2_ particles are exposed on the PTFE matrix, which leads to loose bonding between the PTFE matrix and particles, decrease of crystallinity and weak particle interface. In Figure 13b, except that some particles are exposed on the PTFE matrix, the bonding tightness between the other particles and PTFE matrix is higher than that in Figure 13c, so the mechanical properties of Figure 13b are better than that in Figure 13c. In Figure 13a,d, Al and TiH_2_ particles are tightly embedded in the PTFE matrix. In Figure 13a, the number of voids is more than that in Figure 13d, but the number of voids in both figures is very small. Therefore, the specimen corresponding to Figure 13d has the best mechanical properties due to the tight combination between PTFE matrix and particles inside the specimen.

### 3.5. XRD of the Experimental Specimen before Experiment and Residue Analysis of the Experimental Specimen after Experiment

**Figure 14:**
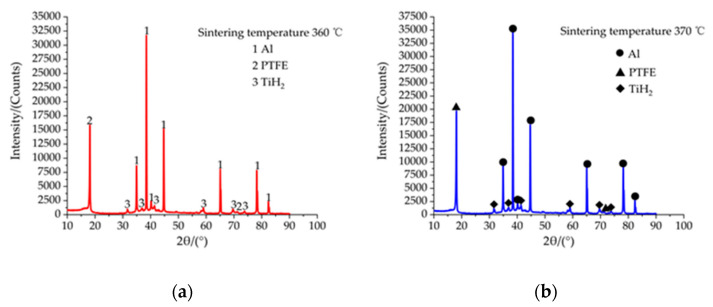
XRD patterns of Al-rich Al/PTFE/TiH_2_ specimens at 360 °C and 370 °C. (**a**,**b**) are XRD patterns of Al-rich Al/PTFE/TiH_2_ specimens at 360 °C and 370 °C, respectively.

In order to verify whether all the samples react in the sintering process, in this paper, the samples with the highest sintering temperature of 370 °C were chosen as the contrast samples at the sintering temperature of 360 °C, and the phase analysis was carried out by means of an X-ray diffractometer (XRD). As can be seen from Figure 14a,b, the diffraction peaks of Al, PTFE and TiH_2_ were detected in the XRD patterns of the specimens sintered at 360 °C and 370 °C, there were no diffraction peaks of other substances and the XRD patterns at the two temperatures were the same. It shows that for all the experimental specimens prepared via mixing, stirring, molding, sintering and other technology no chemical reaction occurred. 

**Figure 15:**
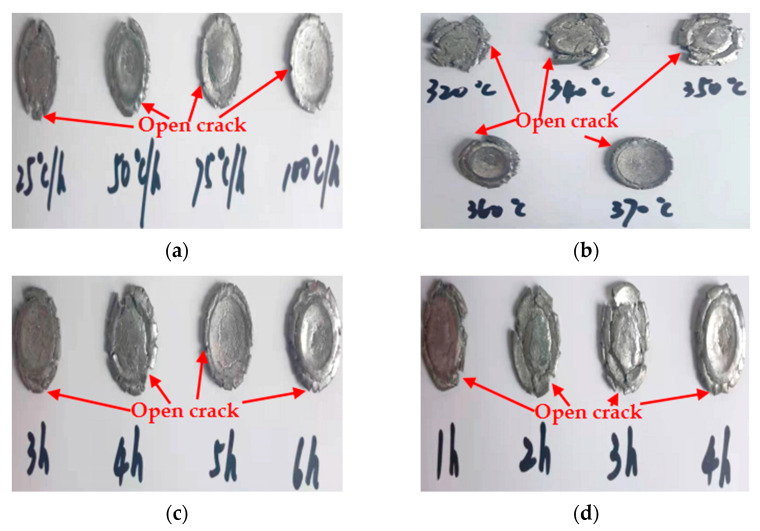
Deformation of static compression experiment specimen with different sintering parameters. (**a**–**d**) show the deformation of specimens with different cooling rates, different sintering temperatures, different holding times at 360 °C and different holding times at 315 °C after the static compression test, respectively.

Deformation of the specimens after the static compression experiment is shown in Figure 15. Figure 15a–d show the deformation of specimens with different cooling rates, different sintering temperatures, different holding times at 360 °C and different holding times at 315 °C after the static compression test, respectively. The deformation degree and ductility of the specimens with different sintering parameters are listed in Table 10. The deformation degree and ductility of the specimen are determined by the failure strain and the maximum true strain, respectively.

As shown in Figure 15, all specimens are fractured and failure destroyed. The analysis is as follows: One is that the outer wall of the specimen propagates along the radial direction, while the crack penetrating the specimen appears in the axial direction, which is caused by the radial tension caused by the axial compression of the material. When the radial tensile force is greater than the tensile limit of the material, microcracks are formed. With the increase of strain, the cracks continue to expand and converge, and finally, a macro axial open crack is formed at the edge. Second, in the process of active material specimen preparation, the combination of PTFE matrix and particles is not tight. When the external force acts on the specimen, the contact between particles is prone to dislocation, resulting in stress concentration, where the energy generated exceeds the energy required for microcrack nucleation, forming microcrack nucleation. After nucleation, the microcracks continue to expand and converge, finally forming the open crack at the edge of the specimen. 

In the process of static compression of all experimental specimens, no bright fire light was found using a high-speed camera. According to Figure 15a–d, the residue after the fracture failure of the specimen is like a saw tooth, there are open cracks at the edge and there is no aggregated carbon black at the open cracks. It is shown that for all the experimental specimens no chemical reaction occurred under quasi-static compression. The reason for this phenomenon may be that the hot spot of ignition is not formed in the specimen during static compression, and the specific reasons may be as follows: (1) The volume of the sealed pores in the specimen is not large enough (the critical condition for the formation of hot spot ignition around the pores is that the pore diameter is 50 μm and the minimum impact force is about 0.1 GPa). (2) When the material is subjected to impact, the material softens more than the material processing hardening effect under the action of fast shear stress, forming plastic deformation of a size less than 1 mm shear band, and cannot reach the condition of the adiabatic shear band hot spot formation. (3) In the process of impact compression, the energy absorbed by the specimen with high toughness converges at the crack tip, and the energy at the crack tip cannot reach the hot spot with enough temperature and size.

## 4. Conclusions

In this paper, the influence of sintering control factors on the mechanical properties of Al-rich Al/PTFE/TiH_2_ active material was studied using a universal energy material testing machine. A scanning electron microscope was used to analyze the morphology. An X-ray diffractometer was used for phase analysis. The compression process of the specimen was recorded using a high-speed camera. The main conclusions drawn are as follows:

(1) With the increase of cooling rate (25 °C/h, 50 °C/h, 75 °C/h, 100 °C/h), the density, yield strength, strain hardening modulus, compressive strength and toughness of Al-rich Al/PTFE/TiH_2_ specimens decrease gradually, the failure strain of the specimens increases gradually, the number of pores inside the specimens increases gradually, the bonding tightness between PTFE and particles decreases, the crystallinity decreases gradually and the crystalline volume shrinkage of PTFE increases gradually. The specimens with different cooling rates have good ductility. According to the above knowledge, the overall mechanical properties of the specimens with a cooling rate of 25 °C are the best.

(2) With the increase of sintering temperature (320 °C, 340 °C, 350 °C, 360 °C, 370 °C), the density, relative density, maximum true strain and toughness of the specimens first increase and then decrease, and the failure strain of the specimens gradually increases. The specimens at the sintering temperature of above 350 °C have good ductility. According to the above knowledge, the overall mechanical properties of the specimens at the sintering temperature of 360 °C are the best. Most of the particles in the samples at the sintering temperatures of 320 °C, 350 °C and 370 °C are exposed on the PTFE matrix, and the bonding between the matrix and particles is not tight, and there are a lot of voids. The PTFE matrix is tightly bonded with the particles inside the specimens at the sintering temperatures of 340 °C and 360 °C, a small number of particles are exposed on the PTFE matrix and there are a small number of voids.

(3) With the increase of holding time (3 h, 4 h, 5 h, 6 h) at 360 °C, the strength and toughness of the material first decrease and then increase. The specimens with different holding times at 360 °C have good ductility. The interface between particles and matrix inside the specimen with a holding time of 6 h is the strongest, and the crack inside the specimen propagation is less, so the overall mechanical properties of the specimen are the best. 

(4) When the sintering time increased from 1 h to 4 h at 315 °C, the compressive strength of the specimen increased by 1.62%, the toughness of the specimen decreased by 0.55% and the failure strain of the specimen decreased by 0.54%. The specimens with different sintering times at 315 °C have good ductility. The strength and failure strain of the specimen with a sintering time of 4 h are the highest. From the microscopic point of view, the bond tightness of PTFE matrix and particles inside the specimens with a sintering time of 4 h is the highest. Based on the above considerations, the overall mechanical properties of the specimen with a sintering time of 4 h at 315 °C are the best.

(5) In the process of quasi-static compression, no chemical reaction occurred for all Al-rich Al/PTFE/TiH_2_ specimens. The main reason for this result may be that no ignition hot spots were formed inside the specimen or it is related to the high content of Al. Therefore, the reactivity of Al-rich PTFE/Al/TiH_2_ materials with 10% content of TiH_2_ under static compression is not significantly affected by sintering control factors.

## Figures and Tables

**Table 1:** Information and mass ratio of components of Al-rich Al/PTFE/TiH_2_ active material.

Main Raw Material Powder	Average Particle Size/(μm)	Purity/(%)	Production Unit	Mass Ratio/(%)
PTFE	27	> 99.5	HS, Guangdong, China	50
Al	6–7	> 99.5	AG, Liaoning, China	40
TiH_2_	4–6	> 99.5	RF, Hunan, China	10
Anhydrous ethanol	/	95	TG, Beijing, China	/

**Table 2:** The sintering parameters shown in Figure 3a.

Figure	Cooling Rate/(°C/h)	Sintering Temperature/(°C)	Holding Time at 360 °C/(h)	Holding Time at 315 °C/(h)
3a	25	360	6	4
	50			
	75			
	100			

**Table 3:** The sintering parameters shown in Figure 3b.

Figure	Ramp rate/(°C/h)	Sintering Temperature/(°C)	Holding Time at 360 °C/(h)	Holding Time at 315 °C/(h)
3b	50	320	6	4
		340		
		350		
		360		
		370		

**Table 4:** The sintering parameters shown in Figure 3c.

Figure	Ramp Rate/(°C/h)	Sintering Temperature/(°C)	Holding Time at 360 °C/(h)	Holding Time at 315 °C/(h)
3c	50	360	3	4
			4	
			5	
			6	

**Table 5:** The sintering parameters shown in Figure 3d.

Figure	Ramp Rate/(°C/h)	Sintering Temperature/(°C)	Holding Time at 360 °C/(h)	Holding Time at 315 °C/(h)
3d	50	360	6	1
				2
				3
				4

**Table 6:** The specific parameters of Figure 6a,c.

Cooling Rate/(°C/h)	Density/(g/cm^3^)	Relative Density ^1^/(%)	Compressive Strength/(MPa)	Failure Strain	Toughness/(MJ/m^3^)
25	2.42923	97.56	82.69	1.611	131.360
50	2.42802	97.51	76.67	1.671	128.103
75	2.42304	97.31	71.68	1.888	120.247
100	2.41944	97.17	63.11	2.165	102.469

^1^ Relative density is the ratio of Density to TMD.

**Table 7:** The specific parameters of Figure 8a,c.

Sintering Temperature/(°C)	Density/(g/cm^3^)	Relative Density/(%)	Compressive Strength/(MPa)	Failure Strain	Toughness/(MJ/m^3^)
320	2.41122	96.84	49.20	0.999	47.291
340	2.42242	97.29	75.77	1.280	91.461
350	2.42429	97.36	63.55	1.745	95.754
360	2.42802	97.51	76.67	1.671	128.103
370	2.41887	97.14	62.07	2.151	104.204

**Table 8:** The specific parameters of Figure 10a,c.

Holding Time at 360 °C/(h)	Density/(g/cm^3^)	Relative Density/(%)	Compressive Strength/(MPa)	Failure Strain	Toughness/(MJ/m^3^)
3	2.43234	97.68	64.38	1.671	115.725
4	2.43353	97.73	59.86	1.421	111.672
5	2.42107	97.23	68.84	1.856	117.964
6	2.42802	97.51	76.67	1.671	128.103

**Table 9:** The specific parameters of Figure 12a,c.

Holding Time at 315 °C/(h)	Density/(g/cm^3^)	Relative Density/(%)	Compressive Strength/(MPa)	Failure Strain	Toughness/(MJ/m^3^)
1	2.42374	97.34	75.43	1.619	128.812
2	2.42172	97.26	66.05	1.680	114.015
3	2.43957	97.97	65.47	1.586	113.222
4	2.42802	97.51	76.67	1.671	128.103

**Table 10:** The deformation degree and ductility of the specimen with different sintering parameters.

Figure	Specimens with Different Sintering Parameters	Degree of Deformation	Ductility
14a	25 °C/h	maximum	worst
	50 °C/h	larger	poor
	75 °C/h	moderate	moderate
	100 °C/h	minimum	best
14b	320 °C	maximum	worst
	340 °C	larger	poor
	350 °C	smaller	moderate
	360 °C	moderate	best
	370 °C	minimum	better
14c	3 h	moderate	moderate
	4 h	maximum	best
	5 h	minimum	worst
	6 h	moderate	better
14d	1 h	moderate	worst
	2 h	minimum	moderate
	3 h	maximum	better
	4 h	smaller	best
